# Circadian Rhythm of Distal Skin Temperature in Healthy Older and Young Women and Its Relationship with Sleep–Wake Rhythm and Environmental Factors under Natural Living Conditions

**DOI:** 10.3390/geriatrics9040102

**Published:** 2024-08-06

**Authors:** Manuela Dittmar, Tina Stark, Stefanie Wedell

**Affiliations:** Department of Human Biology, Zoological Institute, Christian-Albrechts-University, Am Botanischen Garten 9, 24118 Kiel, Germany

**Keywords:** aging, human, circadian rhythm, distal skin temperature, environmental light, sleep-wake timing, physical activity, natural conditions

## Abstract

Little is known about the healthy aging of the circadian timing system under natural living conditions. This study explores changes in the circadian rhythm of distal skin temperature (DST) with aging and relates these changes to sleep–wake timing and environmental influences. DST, sleep–wake timing, 24-h light exposure, and physical activity were measured and averaged over seven consecutive days using temperature sensors, actigraphy with a light meter, and sleep diaries in 35 healthy older women (60–79 years) and 30 young women (20–34 years). Circadian rhythm characteristics, describing strength (amplitude) and timing (acrophase) of the DST rhythm, were calculated using cosinor analysis. The older adults displayed an 18–19% smaller amplitude and a 66–73 min earlier acrophase (peak time) for DST rhythm than the young adults, indicating a weaker and phase-advanced DST rhythm. The phase advance for DST was not due to an earlier evening increase, but to a shorter nocturnal plateau period. Daytime light exposure inversely affected strength (amplitude) but not phasing of the DST rhythm in older adults. The DST rhythm was 3.5 times more advanced than the sleep–wake rhythm, showing an altered phase relationship (phase angle) between both rhythms with aging. The phase angle was more heterogeneous among older adults, showing differential aging. The phase advance for DST rhythm and the altered and heterogeneous phase relationship between DST and sleep–wake rhythms were not related to ambient light exposure and the physical activity of older adults. This suggests that healthy aging of the circadian system might be due to endogenous mechanisms such as an internal rearrangement rather than external influences.

## 1. Introduction

Many human physiological and behavioral traits follow circadian rhythms that exhibit a maximum value (peak) and a minimum value (nadir) within a period of approximately 24 h. Circadian rhythms are endogenously generated, self-sustained by molecular circadian clocks [1], and entrained to 24 h by external cues such as environmental light. The strength and timing of a circadian rhythm is described by its amplitude and acrophase (timing of peak), respectively. A less robust circadian system, as reflected by a flatter amplitude and an earlier acrophase of circadian rhythms, accompanies human aging [2,3,4,5,6,7]. The earlier acrophase indicates a phase advance that may arise from a shortening of the circadian period length. Age-related circadian phase advances have been demonstrated for several human traits including core body temperature (CBT) rhythm, melatonin rhythm, and sleep–wake rhythm [8,9,10,11,12]. Likewise, a reduction in the circadian amplitude has been observed for human traits with aging, e.g., for the melatonin rhythm, the rest–activity pattern, and the CBT rhythm [11,13,14,15,16].

Changes in human temperature rhythms with aging are of special interest because the thermoregulatory system is coupled with sleep initiation [17,18]. Most studies in older people have examined the circadian rhythm for CBT [5,10,12,16], while little is known on the circadian rhythm for distal skin temperature (DST) [19]. DST refers to the temperature at distal skin areas on the hands and feet and has been considered as a major endogenous regulator of the CBT rhythm [20]. The circadian variation in DST is involved in the regulation of sleep–wake timing. The nocturnal vasodilatation of arteriovenous anastomoses of blood vessels in distal skin areas increases DST, leading to distal heat loss and thereby promoting the onset of nocturnal sleep [21]. Since sleep timing is coupled with the thermoregulatory system, age-related changes in the rhythm of DST may influence sleep initiation [17].

Furthermore, the DST rhythm has been proposed as a general marker for the circadian timing system in humans [22,23]. So far, the circadian pattern for DST has been examined in young and middle-aged adults under laboratory and ambulatory conditions [23,24,25,26], while little is known on the circadian DST rhythm of healthy older adults [19]. Harfmann and colleagues [27] examined the DST rhythm in a mixed group of middle-aged and older adults (50–70 years) with and without metabolic syndrome and diabetes. The study was restricted to overweight and obese adults and analyzed the amplitude but not the acrophase of the DST rhythm. The acrophase is the time at which the peak of the circadian DST rhythm occurs. In the context of healthy aging, the acrophase is an important parameter because a shift in the acrophase may result in a desynchronization between the DST rhythm and other circadian rhythms, for example, between the DST rhythm and the sleep–wake rhythm. However, the temporal relationship (phase angle) between the acrophase of the circadian DST rhythm and the sleep-onset time of the sleep–wake rhythm has not yet been analyzed in healthy older people under natural living conditions. Previous studies in older adults have focused on the relationship between sleep–wake timing and the CBT rhythm [10]. Another study analyzed the effect of season on sleep and skin temperature in healthy older adults, but the study was restricted to nighttime temperature data and did not analyze the circadian rhythm of DST [28].

Several mechanisms are under discussion that may explain the weaker circadian timing system observed in older adults. Firstly, an age-related deterioration in the function of the central circadian pacemaker, the suprachiasmatic nuclei (SCN) in the hypothalamus [29,30,31], controlling circadian rhythms, might be responsible for age-related alterations. This could also affect the internal synchronization between different circadian rhythms. Second, an altered responsiveness in the central circadian pacemaker to environmental photic stimuli might be causal [32,33,34]. A further explanation is a lowered sensitivity of older adults to solar light due to changes in eye anatomy and physiology such as lens yellowing, pupillary miosis, and cataracts [35,36]. This might disturb the entrainment of circadian rhythms with environmental zeitgebers during aging.

Environmental influences, primarily the light–dark cycle, synchronize endogenous circadian rhythms to a 24-h day. Although environmental cues may mask the circadian rhythm for DST under natural living conditions, Martinez-Nicolas et al. [25] reported in young adults a similar circadian pattern for the DST rhythm under masked and unmasked study conditions. The authors noted that environmental influences affected the amplitude of the DST rhythm, while the acrophase and mesor were more stable and robust parameters of the circadian rhythm. The authors measured the rhythm of DST at the wrist (hand) of young adults. Because hands are more exposed than feet to environmental influences such as light and temperature, it would be interesting to further examine the circadian rhythm of foot temperature. The circadian DST rhythms of the foot and hand have not yet been compared in healthy older adults under natural circumstances. This would be particularly interesting because older people are less affected by environmental factors than younger people [37].

The present study focuses on healthy aging of the circadian rhythm for DST and its relationship with sleep–wake timing and environmental influences. The study was conducted under natural living conditions because they represent the real-life situation of older people. For investigating normal aging processes, a well-defined sample of healthy older adults aged ≥60 years was recruited and compared with a young adult control group aged <35 years. To show clear age group differences, middle-aged adults (40–59 years) were not included in accordance with the results of Júnior and Sardeli [38]. Because the circadian rhythm of DST refers to both hand and foot skin temperature, a comparison of both skin areas of the body was carried out. This would provide information on whether aging might differently affect DST at the hand and foot areas because there is evidence from healthy young adults that exogenous melatonin increases the DST on hands but not on feet [39]. The hormone melatonin can induce vasodilation in arteriovenous anastomoses in distal skin areas [20]. The first aim of the present study was therefore to compare the circadian rhythm for DST on the hand and on foot in healthy older adults, relative to young adults. In addition, there is evidence that aging is accompanied by an altered circadian phase relationship (phase angle) between endogenous circadian rhythms and the sleep–wake cycle [40]. So far, there is no literature on the circadian phase angle between the acrophase of the DST rhythm and the timing of the sleep–wake cycle from healthy older adults under real-life conditions. The second aim of this study was therefore to analyze the circadian phase relationship between the acrophase of the DST rhythm and the timing of the sleep–wake rhythm in healthy older adults, relative to young adults. This would provide knowledge on whether a desynchronization or rearrangement between the DST rhythm and the sleep–wake rhythm may accompany normal aging. Possible mechanisms for an altered phase relationship between circadian rhythms are endogenous processes (alterations of the circadian timing system), exogenous influences (for example, environmental light exposure), or a combination of both. Experimental conditions have shown that the application of bright light at night following a shift in the timing of sleep phases delayed the rhythm of skin temperature in young adults [22]. It remains unclear whether this would also occur under natural living conditions in older adults. The third aim was to examine in healthy older and young adults the effect of external influences on the circadian DST rhythm itself and on the circadian phase relationship between the DST rhythm and the sleep–wake rhythm. External influences on the DST rhythm were the overall daytime and nighttime light exposure, adapted to the individual bedtimes of the participants, as well as their overall physical activity, all examined and averaged over a period of seven days.

## 2. Materials and Methods

### 2.1. Participants

Participants were 65 healthy volunteers comprising 35 older women (age range 60–79 years, mean age ± SD, 68.9 ± 4.9 years) and 30 young women (20–34 years, 25.6 ± 3.6 years) from the same geographic area in northern Germany. The older women lived independently in their own households. The participants were recruited through advertisements in notice-board postings, local newspapers, and university lectures for older or young people. The study was restricted to the female population because sex differences have been reported in rhythm analysis for CBT, showing that women displayed a phase-advanced acrophase in body temperature compared to men [41]. There were strict criteria for inclusion in the study to ensure that results are due to normal aging processes and not to secondary effects such as diseases or medication. Inclusion criteria were a normal sleep–wake pattern as well as absence of chronic and acute disorders and medication, as determined by medical history. Women using melatonin, which could affect circadian rhythms, were not included in the study. Further exclusion criteria were smoking, underweight (BMI < 18.5 kg/m^2^), obesity (BMI > 29.9 kg/m^2^, except for one older woman having a BMI of 31.5 kg/m^2^), as well as shift work and transmeridian travel within three months prior to the study. All older women were postmenopausal. Eighteen of the thirty young women were taking oral contraceptives. The ethics committee at the Medical Faculty of the Christian-Albrechts-University, Kiel, provided approval of the study protocol (A 116/12), and each study participant gave written informed consent prior to data collection.

### 2.2. Study Design

All participants were personally instructed to maintain their regular lifestyle and sleep schedules at their usual times one week before and during ambulatory data collection. In addition to a detailed verbal instruction, each participant received an information brochure to take home. The period of ambulatory data collection comprised one week (7 × 24 h) during which DST rhythm, sleep–wake rhythm, physical activity, and light exposure were consecutively monitored using a wireless temperature data logger and an actigraph with integrated light meter. In addition, all participants completed a daily sleep–wake diary during this period. They were instructed to stay at home from 18:00 h until bedtime, thus being exposed in the evening to ordinary indoor room light but not to outdoor sunlight. They were also asked not to take a shower, bath, or footbath from 18:00 h until awakening to prevent external influence on skin temperature.

### 2.3. Assessment of General Characteristics

Anthropometric characteristics of the participants, i.e., body height, body weight, and body circumferences (waist, hip) were measured in light indoor clothes without shoes in standing position to the nearest 0.1 cm or 0.1 kg, using anthropometer, electronic scale (TGF 302H, Rossmann, Burgwedel, Germany), and tape measure, respectively. The body mass index (BMI) was calculated by dividing weight (kg) by height squared (m^2^). The waist-to-hip index was calculated by dividing waist circumference (cm) by hip circumference (cm). Body composition (fat mass, lean body mass, body cell mass) and basal metabolic rate were determined in the morning after an overnight fast using a whole-body tetrapolar bioimpedance analyzer (Nutriguard-M, Data Input, Darmstadt, Germany) and Nutri-Plus 5.1 software of the manufacturer of the bioimpedance analyzer [42].

### 2.4. Recording of Distal Skin Temperature

Skin temperature readings were continuously monitored every 10 min over seven consecutive days using a wireless temperature data logger (Thermochron iButton^®^ DS1921H-F5, Maxim, Dallas, TX, USA). This device measures and stores skin temperature data in a temperature range between +15 °C and +46 °C. It has a sensitivity of 0.125 °C and has been shown to be a reliable and valid measure of human skin temperature [43]. The device (diameter × height: 16 × 6 mm^2^) contains a temperature sensor, a computer chip with a real-time clock memory, and a 3V lithium battery. The temperature sensor and battery are located towards the top side and the bottom side of the iButton, respectively. The iButtons were positioned on the non-dominant side of the body with the top side over the skin because this side has a faster response to thermal changes [44]. They were placed over the radial artery of the inside of the wrist [23] and over the inner calcaneus bone of the heel of the foot [45]. They were fixed to the skin with adhesive surgical tape (Leukosilk, BSN Medical GmbH, Hamburg, Germany) and isolated from environmental temperature using a cotton sport band and a sock at the wrist and heel, respectively. The temperature data stored in the iButton were transferred through an adapter (DS9490R; Maxim, Dallas) to a personal computer using iButton OneWireViewer software version 1.4, which was provided by the manufacturer of the iButton.

### 2.5. Assessment of Sleep–Wake Variables, Physical Activity, and Light Exposure

Sleep–wake variables, physical activity pattern, and light exposure were objectively measured by actigraphy. Participants wore an actigraph-type Actiwatch^®^ 2 (AW2, Phillips Respironics, Murryville, PA, USA) on the wrist of their non-dominant arm [46] for a period of seven consecutive 24-h days. A 7-day average was calculated for each variable. The actigraph is an accelerometer that utilizes a piezo-electric sensor as the activity-sensing element. Based on the activity of the participants, the accelerometer produced voltage, which was converted into activity counts, recorded in 1 min epochs [47,48,49]. For each study day, participants were instructed to press an event marker on the actigraph when they intended to fall asleep in the evening and again when they awoke in the morning. After seven days, participants returned the actigraph. Data were downloaded onto a computer and scored using Actiware 6.0.1 software which was supplied by the manufacturer.

Actigraphy was used to determine sleep and wakefulness by the software algorithm. Sleep data from actigraphy correlated highly with data derived from polysomnography [50]. Derived sleep variables were awakening time, sleep-onset time, sleep-onset latency (SOL; time needed to fall asleep), total sleep time (TST; sleeping time excluding periods of wakefulness), wake time after sleep-onset (WASO), and sleep efficiency (SE; percentage of time in bed spent sleeping). Actigraphy was combined with sleep-log data, as has been suggested by Ancoli-Israel and colleagues [47]. Along with the actigraph, participants completed for the seven days a daily sleep–wake diary using the morning/evening protocol of the German Sleep Society (DGSM) [51]. In the evening section of the protocol, they noted their bedtimes, lights-off time before sleep, and duration of daytime naps. In the morning section of the protocol, they noted wake-up time (lights on), get-up time, periods of wakefulness during the night, and sleep-onset latency. Data from the actigraphy and sleep diary were compared to detect discrepancies among the actigraphic event marker, activity pattern, and sleep diary. The diary allows differentiating awake motionless periods from true sleep periods, thereby improving the accuracy of sleep-onset identification [52]. In addition, it allows for manually adding data to the data file when participants forgot to press the event marker on the actigraph.

Actigraphy was further used to measure the rest–activity level of the participants. Total activity, defined as the sum of activity counts for all epochs, was determined for wake time (daytime; defined as rise time to bedtime) and for sleep time (nighttime; defined as bedtime to rise time), using for each variable the average of seven days of data collection.

In addition, the actigraph was used to measure white light exposure at 1 min intervals over the period of seven days using an integrated light meter. Participants were instructed to ensure that the light sensor was not covered by clothing. Actiware software was used to calculate from measured data the average light intensity (Lux), defined as the average of valid light data for all epochs from the start time to the end time of the given interval. It was calculated separately for wake time (daytime) and sleep time (nighttime), based on the average of seven days of data collection for each variable.

### 2.6. Statistical Analyses

Circadian rhythm characteristics of DST data were calculated by cosinor analysis using Chronos-Fit software version 1.06 [53]. This rhythm analysis combines partial Fourier analysis and stepwise regression technique, fitting each harmonic separately and checking significance by F-tests. Principal rhythm characteristics calculated were mesor (average value of temperature rhythm fitted to a cosine function), amplitude (distance from the mesor value to the peak value of the cosine function), and acrophase (timing of the peak value fitted to a cosine function relative to local 00:00 h), describing 24-h rhythm-adjusted mean, strength, and timing of DST rhythm, respectively. As a measure of goodness-of-fit, the %rhythm was calculated, describing the percentage of overall data variation accounted for by the fitted cosine curve [54]. Chronos-Fit software was also used for graphic presentation of group means at 10 min intervals with 95% confidence limits over a period of 24 h.

Statistical analyses were performed using SPSS software package for MS Windows, release 21 (IBM, Armonk, NY, USA). Results are expressed as mean ± standard deviation (SD) or standard error of the mean (SEM). Normality of metric data was tested using Kolmogorov–Smirnov test. Group differences in general characteristics between older and young women were tested using independent samples *t*-tests. Reproducibility of DST over seven days and differences between age groups were tested for each circadian variable (mesor, amplitude, and acrophase) by analysis of variance (ANOVA) for repeated measures with the inner-subject factor (time) and between-subject factor (age group). To characterize the circadian phase relationship between DST rhythm and sleep–wake rhythm, phase angles (PA) were calculated as the difference between the sleep-onset time of the sleep–wake rhythm and the acrophase of the DST rhythm. Sleep-onset time was used as a measure for sleep timing following Sletten and colleagues [55]. Correlation analysis was performed to determine the relationship between DST, sleep times, physical activity, and environmental light exposure using Pearson or Spearman rank correlation coefficients. A two-tailed *p*-value of <0.050 was considered statistically significant.

## 3. Results

### 3.1. General Characteristics and Sleep–Wake Rhythm of Participants

Table 1 summarizes general characteristics of the study participants. Older women displayed a significantly higher BMI and waist circumference, but lower body cell mass and basal metabolic rate than young women. During the seven days of recording, overall physical activity and light exposure were not significantly different between older and young women. Both age groups showed only minimum movement and light exposure during the sleep episode at night but higher physical activity and light exposure during daytime, indicating normally entrained sleep–wake rhythms. Older compared with young women displayed phase-advanced sleep–wake timing, as indicated by a 60 min earlier awakening time (*p* < 0.001), 30 min earlier bedtime (*p* = 0.001), and 21 min earlier sleep-onset time (*p* = 0.045). Furthermore, older women had a 10 min longer sleep-onset latency (*p* = 0.027), 47 min shorter total sleep time (*p* = 0.001), and 8% smaller sleep efficiency (*p* < 0.001) than young women, indicating poorer sleep quality in this age group.

### 3.2. Circadian Rhythm of Distal Skin Temperature of Hand and Foot in Older and Young Participants

Cosinor analyses showed highly significant circadian rhythms for DST in the overall fits for each woman for each of the seven days. This was observed for both older women (hand: *p* = 0.0001 to 0.1 × 10^−11^; foot: *p* < 0.1 × 10^−5^ to 0.3 × 10^−8^) and young women (hand: *p* = 0.4 × 10^−4^ to 0.1 × 10^−11^; foot: *p* < 0.1 × 10^−5^ to 0.1 × 10^−10^). Measurements of mesor, amplitude, and acrophase of the DST rhythm were reproducible over the seven days in both age groups (ANOVA, each *p* > 0.050, Table 2). Table 2 presents mean values for the circadian mesor, amplitude, and acrophase of DST, averaged over seven days for each age group. Differences between age groups were larger for the parameters of foot temperature rhythm than those for hand temperature rhythm. Older women had a slightly smaller circadian mesor (non-significant), a weaker amplitude (*p* < 0.050), and an earlier acrophase (*p* < 0.010) compared to young women for both hand and foot DST rhythm. The mean amplitude was reduced in older women by 0.28 °C (19.4%) for hand DST (*p* = 0.046) and by 0.40 °C (18.2%) for foot DST (*p* = 0.023), when compared to young women. The mean acrophase was advanced in older women by 1.10 h for hand DST rhythm (*p* = 0.004) and by 1.22 h for foot DST rhythm (*p* = 0.001) compared to young women. In both age groups, the acrophase for foot DST rhythm occurred approximately three hours earlier than the acrophase for hand DST rhythm. The earlier acrophase of older women was not related with their reduced amplitude (hand: r = −0.07, *p* = 0.710; foot: r = 0.10, *p* = 0.588).

**Figure 1:**
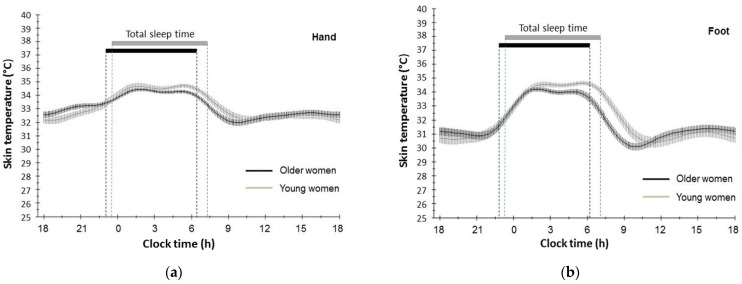
Circadian rhythm of distal skin temperature of hand (**a**) and foot (**b**), recorded at 10 min intervals over a five-day period in 65 healthy participants (35 older and 30 young women). Data points are mean fitted values and 95% confidence limits, calculated with Chronos-Fit software. Black and grey horizontal bars represent mean total sleep time in older and young women, respectively.

The DST rhythm data of the hand and foot were graphically fitted for each age group. Due to the quantity of data, the software was restricted to simultaneously processing data from five days, but not from all seven days. The chronogram in Figure 1 shows that both older and young women displayed a typical circadian pattern in DST over 24 h, with highest temperature values observed at night. The temperature pattern of older and young women shows an increase in DST in the evening before bedtime, a high temperature plateau at night, and a decrease in the morning. An additional smaller temperature maximum occurred around 16:00 h. This pattern was observed for both hand and foot DST rhythm. Further examination of the chronograms showed that the earlier acrophase for foot DST rhythm in older women was not due to an earlier evening increase in foot temperature, but rather to a shorter nocturnal plateau period and an earlier morning decrease.

The acrophase for DST rhythm in older women did not significantly correlate with their total sleep time (hand: r = 0.20, *p* = 0.302; foot: r = −0.07, *p* = 0.707). The same was noted in young women (hand: r = 0.07, *p* = 0.746; foot: r = −0.03, *p* = 0.896). Neither the acrophase nor amplitude of the DST rhythm were related to indicators of sleep quality such as sleep-onset latency, total sleep time, and sleep efficiency (*p* > 0.050) in older and young women.

### 3.3. Phase Relationship (Time Interval) between Distal Skin Temperature Rhythm and Sleep–Wake Rhythm

**Figure 2:**
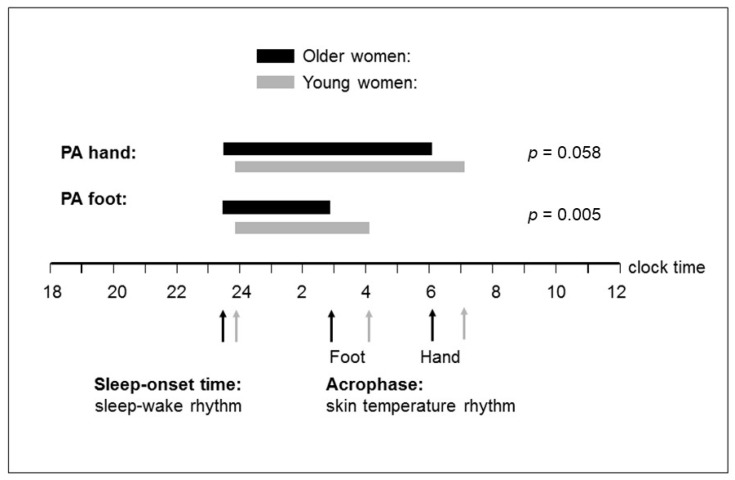
Phase relationship between the acrophase (peak time) of distal skin temperature (DST) rhythm and sleep-onset time of sleep–wake rhythm, averaged over seven consecutive days. Black arrows (older women) and grey arrows (young women) represent the time points of the sleep-onset time and the acrophase of DST rhythm. The sleep-onset time was 21 min and the acrophase for DST rhythm was approximately 70 min advanced in the older women relative to young women. Black (older women) and grey horizontal bars (young women) represent the mean phase angle (PA, time interval) between the sleep-onset time and the acrophase of DST rhythm, separately for hand DST and foot DST. The mean PA of older women was shorter by approximately 50 min compared with young women, both for hand DST (*p* = 0.058) and foot DST (*p* = 0.005). This indicates that the circadian rhythm for DST is phase-advanced relative to the sleep–wake rhythm in older women by approximately 50 min, when compared with young women.

**Figure 3:**
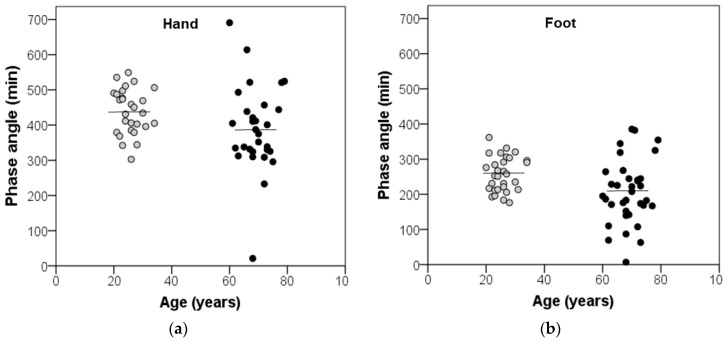
Variation in the phase angle, calculated as the time interval between the sleep-onset time of the sleep–wake cycle and the acrophase of the distal skin temperature rhythm of (**a**) hand and (**b**) foot, averaged over seven consecutive days in 35 healthy older women and 30 young women. Horizontal lines indicate mean values. The variation in the phase angle is greater among the older women (black circles) than among the young women (grey circles); foot: *p* = 0.014; hand, *p* = 0.055.

Since the phase advance of DST rhythm in older women may stem from their earlier phase of the sleep–wake rhythm, a phase angle was calculated to describe the relative timing between both rhythms. It was calculated as the difference in time between sleep-onset time of the sleep–wake rhythm and the acrophase of the DST rhythm. The mean phase angle ± SEM between the sleep–wake rhythm and hand (foot) DST rhythm was 389 ± 22 min (206 ± 15 min) in older women and 439 ± 12 min (257 ± 9 min) in young women. This means that the phase angle (time interval) between hand (foot) DST rhythm and sleep–wake rhythm was 50 (51) min shorter in older women than in young women. Thus, the DST rhythm of the older women was more phase-advanced relative to the sleep–wake cycle than it was the case in young women (Figure 2). The observed difference in phase angle between both age groups was statistically significant for foot DST rhythm, but not for hand DST rhythm (foot: t = −2.92, *p* = 0.005; hand: t = −1.93, *p* = 0.058). In addition, the inter-individual variation in the phase angle was greater among the older women than among the young women (foot: F = 6.41, *p* = 0.014; hand: F = 3.84, *p* = 0.055; Figure 3). The individual phase angles of the older women ranged from 21 to 691 min with respect to hand DST and from 7 to 385 min with respect to foot DST. In young women, individual phase angles ranged from 303 to 549 min for hand DST and from 176 to 362 min for foot DST.

### 3.4. Relationship of Distal Skin Temperature Rhythm and Phase Angle with the Environmental Light Exposure and Physical Activity

Table 3 displays the correlation coefficients and *p*-values regarding the relationship of amplitude and acrophase for hand and foot DST rhythm with environmental light exposure, physical activity, and sleep–wake timing during the seven days of data recording. The circadian amplitude of both hand and foot DST rhythm was inversely and significantly related to daytime light exposure in the older women (hand: *p* = 0.023; foot: *p* = 0.007), but not in the young women. Neither in the older women nor in the young women was the amplitude of DST rhythm related to nighttime light exposure. The circadian acrophase of the DST rhythm of the hand and foot was neither related to daytime light exposure nor to nighttime light exposure in any age group. Neither the amplitude nor the acrophase for DST rhythm of the hand and foot was associated with the level of physical activity in the older and young women.

The acrophase of the DST rhythm was not related to sleep–wake times in the older women but was positively and significantly related in young women (awakening time, hand: *p* = 0.031, foot: *p* = 0.002; sleep-onset time, hand: *p* = 0.050, foot: *p* = 0.001; Table 3). The sleep–wake timing of the older women was not related to environmental factors. The earlier sleep-onset and awakening times of the older women did not correlate with their physical activity (r = −0.02, *p* = 0.920; r = 0.05, *p* = 0.760) nor light exposure (r = −0.15, *p* = 0.381; r = −0.24, *p* = 0.161).

Table 3 also shows the correlation coefficients of the phase angle with environmental factors. The phase angle between DST rhythm and sleep–wake rhythm did not significantly correlate with daytime light exposure, nighttime light exposure, daytime physical activity, or nighttime physical activity, neither in older women nor in young women.

## 4. Discussion

### 4.1. Changes in the Amplitude for Circadian DST Rhythm with Healthy Aging and Environmental Influences

This study examined the circadian rhythm for DST in healthy older and young adults under natural living conditions. The general 24 h pattern for the DST rhythm corresponds in both age groups to that described in adults of ambulatory and laboratory studies showing a maximum DST at night and a minimum DST in the daytime [19,22,24]. Both older and young women displayed significant circadian rhythms for hand DST and for foot DST. However, the mean percentage of overall data variation accounted for by the fitted cosine curve (%rhythm) was higher for foot DST rhythm (83–85%) than for hand DST rhythm (67–69%) in both age groups. This indicates a better goodness-of-fit for foot DST than for hand DST rhythm data.

The older women displayed a significant amplitude reduction for the DST rhythm, compared to young women, indicating a weaker rhythm for DST with normal aging. The magnitude of amplitude reduction was similar for hand DST rhythm (18.2%) and foot DST rhythm (19.4%), suggesting that hand and foot temperature may lead to comparable results in healthy older adults. For the circadian amplitude of foot DST rhythm, no comparative literature data are available from healthy older adults under natural living conditions, while the smaller amplitude for hand DST rhythm agrees with previous results showing a trend towards flatter amplitude in wrist DST rhythm with aging [19]. Another study reported for a mixed group of middle-aged and older adults, who were all overweight or obese, a slightly lower mean amplitude for wrist DST [27] than we observed in the healthy and on average normal-weight older women. The authors further noted a reduced amplitude for wrist DST in patients with metabolic syndrome. Nevertheless, the amplitude reduction among the present older women was not caused by diseases because only healthy women were included in this study. It can therefore be assumed that their flatter amplitude results from normal aging processes. A possible mechanism is the known age-related decrease in skin blood flow and the diminished ability of aged skin to vasodilate during the night, when vasodilation in hands and feet is high [56]. This would attenuate the nocturnal increase of DST, thereby reducing its circadian amplitude [57].

To the best of our knowledge, this is the first study that assessed in healthy older adults the influence of 24 h daytime light exposure on the circadian amplitude for hand and foot DST rhythm under real-life conditions. We found that the overall daytime light exposure affected strength (amplitude) but not phasing (acrophase) of the DST rhythm in the older women. This finding is consistent with results in young adults showing that environmental influences, including light exposure, most affected the amplitude of the DST rhythm, whereas the acrophase was a more stable parameter for characterizing the circadian rhythm [25]. The older adults displayed a significant and inverse relationship between daytime light exposure and circadian amplitude for the DST rhythm on both hand and foot regions. This fits with the results of a forced desynchrony laboratory study in young adults showing that exposure to bright white light significantly decreased the circadian amplitude of skin temperature [58]. However, we observed no association between light exposure and amplitude for DST in the young group, although the magnitude of daytime light exposure did not differ between both age groups. A possible explanation might be an altered responsiveness of the circadian pacemaker to environmental photic stimuli in older adults [32,33].

### 4.2. Changes in the Acrophase for Circadian DST Rhythm with Healthy Aging and Environmental Influences

This study found a significant phase advance of the DST rhythm in healthy older women relative to young women. The mean phase advance was similar for hand DST rhythm (66 min) and for foot DST rhythm (73 min), but the variation in acrophase was higher for hand DST compared to foot DST. The earlier acrophase of older women was not related to their flatter amplitude. This applies to both hand and foot DST rhythm. There is no comparative literature data for foot DST rhythm from older adults, but the present findings confirm results of a significant phase advance for wrist DST rhythm with aging [19]. A comparison of hand and foot DST rhythms revealed that the acrophase for foot DST rhythm occurred approximately three hours earlier than the acrophase for hand DST rhythm. Since this was observed in both age groups, it cannot be attributed to aging processes. Rather, it indicates that the acrophases for hand and foot DST rhythm should not be used alternatively when the absolute timing of the circadian DST rhythm is examined.

Several possible mechanisms may explain the advanced phase for the DST rhythm in the older women. Firstly, a shorter period of the endogenous oscillator might cause the phase advance. In support of this, we found that the earlier acrophase for the DST rhythm of the older women was not due to an earlier evening increase of DST, but rather to a shorter nocturnal plateau length resulting in an earlier morning decrease of DST, when compared to young women (cf. Figure 1). The shorter nocturnal plateau could therefore arise from a shortening of the circadian period length of the DST rhythm with aging. This might also explain why the advanced circadian acrophase of the DST rhythm was not related to the shorter total sleep time in the older women, contrary to the young women. The shorter nocturnal plateau length was more pronounced for foot DST rhythm than for hand DST rhythm. A possible explanation is the higher percentage of overall DST data variation accounted for by the fitted cosine curve (%rhythm) for foot DST compared with hand DST. Thus, foot DST data may provide more information on healthy aging of the circadian DST rhythm than hand DST data does.

A second mechanism for the advanced DST rhythm observed among the older women could be a reduced exposure to synchronizing external stimuli (zeitgebers) with aging, in particular, to environmental light. However, this does not apply to the present older women because their overall daytime and nighttime light exposure did not differ from that in young women during the seven days of examination. A third mechanism might be an altered responsiveness to zeitgebers such as environmental light in older adults [59]. Nevertheless, the acrophase of the DST rhythm of older women was not related to their overall daytime and nighttime light exposure during the seven days of examination. The same was found for young women. This is in line with the results of a laboratory study in which a bright light exposure phase delayed the melatonin rhythm to the same extent in older subjects as in younger subjects [60]. In addition, we found that neither the acrophase for the DST rhythm nor the earlier sleep-onset and awakening times of the older women were related to their overall environmental light exposure and physical activity. This argues against environmental factors as a primary reason for the observed phase advance in DST in the older women. It seems, therefore, more likely that endogenous mechanisms such as a shortening of the circadian pacemaker are responsible for the age-related advance in DST. Further evidence comes from animal studies that demonstrated a shorter circadian free-running period of the SCN with aging [61]. Taken together, the present findings suggest that endogenous mechanisms, such as alterations of the central pacemaker with aging, rather than environmental influences might cause the phase advance in DST with normal aging.

### 4.3. Altered Phase Relationship between Circadian DST Rhythm and Sleep–Wake Rhythm with Aging

Yamazaki and colleagues [61] supposed, based on animal studies, that aging may act primarily on interactions among circadian oscillators. We were therefore particularly interested in the phase relationship between the DST rhythm and the sleep–wake rhythm. We found that both rhythms were phase-advanced in the healthy older women relative to the young women. The earlier habitual bedtimes, sleep-onset times, and awakening times in the older women confirm previous studies [9,10,62]. The mean phase advance in older women was approximately 70 min for the DST rhythm and 21 min for the sleep–wake rhythm. This means that the DST rhythm was 3.5 times more phase-advanced than the sleep–wake rhythm in older relative to young women. This indicates an altered phase relationship between both rhythms with aging, characterized by a significant phase advance of the DST rhythm relative to the sleep–wake rhythm. Thus, sleep occurred in the older women at a later biological time than in young women, despite earlier bedtimes in the older women. The altered phase relationship between both rhythms was similar for hand and foot DST rhythms (50 and 51 min), but it was only significant for foot DST rhythm, possibly due to the higher %rhythm for foot DST. Therefore, it might be more useful to examine foot DST instead of hand DST in the context of healthy aging.

While young women displayed a positive and significant association between the acrophase of their DST rhythm and sleep–wake times, this was no longer the case for older women. This may indicate a weaker relationship between DST rhythm and sleep–wake timing with aging. A possible mechanism is that aging affects the internal synchronization between the DST rhythm and the sleep–wake rhythm. If both rhythms are separate oscillatory systems, their endogenous synchronization by the circadian pacemaker (SCN) and/or their external synchronization with so-called zeitgebers (ambient light) may change with aging. However, we found that the altered phase relationship observed between the DST rhythm and sleep–wake rhythm with aging was not related to the overall environmental light exposure or physical activity of the older women. This suggests that endogenous mechanisms rather than external influences might be responsible for the age-related change in the phase relationship between DST rhythm and sleep–wake rhythm. A possible endogenous mechanism would be an internal circadian rearrangement of the circadian timing system during aging, caused by the known changes in the morphology and chemistry of the circadian pacemaker with aging [63]. Thus, aging of the circadian pacemaker may lead to an internal circadian rearrangement or to a misalignment between the timing of the sleep–wake rhythm and the DST rhythm. Evidence for an age-related rearrangement of internal phase angles with aging, but not for a circadian desynchronization with aging, comes from a laboratory study that investigated circadian rhythms of melatonin and CBT in older and young adults [64]. Future studies are necessary to elucidate possible mechanisms for the altered phase relationship observed between the DST and sleep–wake rhythms with aging.

We further found among the older women a greater heterogeneity across the phase relationship between the DST rhythm and the sleep–wake rhythm, when compared to young women. The heterogeneity was not related to the environmental light exposure and physical activity of the older women. Therefore, the greater heterogeneity in phase relationship might rather reflect inter-individual differences in physiological aging processes of the circadian timing system. Differential aging is already known to occur in many human traits [65]. In this study, the main criteria for defining the health of the participants were absence of disease and medication. This definition of health did not take into account metabolic factors such as levels of lipids (triglycerides, LDL, and HDL), blood glucose level, blood pressure, and dietary patterns. It is known that the aging process is associated with changes in metabolism [66,67] and that many metabolic parameters have circadian rhythms [68]. Recent studies have reported age-related changes in the circadian regulation of metabolic parameters in humans [69] and animals [70]. Therefore, the observed heterogeneity in phase angle between the DST rhythm and the sleep–wake rhythm among the older participants could reflect a variation in metabolic and other parameters. If endogenous factors are responsible for the observed differences between young and older women, a more rigorous and complex definition of healthy aging is required for future studies to control for these factors. Future studies should address differential aging to better understand physiological aging processes of the circadian timing system.

## 5. Conclusions

This study demonstrated significant circadian rhythms for hand DST and foot DST in older and young women under real-life conditions. A comparison of hand and foot DST rhythms revealed (a) that measurements for foot DST produced better cosine fits, (b) that age group differences in foot DST were more pronounced, and (c) that the acrophase for foot DST rhythm occurred in both age groups approximately three hours earlier than the acrophase for hand DST. This suggests using foot DST rather than hand DST when investigating healthy aging. Older women showed a weaker (flatter amplitude) and advanced (earlier acrophase) circadian rhythm for DST than that of young women. The earlier acrophase of older women was not due to an earlier evening increase in DST, but to a shorter nocturnal plateau length, resulting in an earlier morning decrease of DST. The shorter nocturnal plateau could arise from a shortening of the circadian period length for DST rhythm with aging. The main finding of this study was that in older women, the DST rhythm was 3.5 times more advanced than the sleep–wake timing, indicating an altered circadian phase relationship between both rhythms with normal aging. In line with this, the acrophase of the DST rhythm was not related to sleep–wake times in the older women but was positively and significantly related in young women. Furthermore, the phase relationship between the DST rhythm and the sleep–wake rhythm was more heterogeneous among the older women, indicating differential aging of the circadian timing system. In older women, the amplitude of the circadian DST rhythm was inversely related to their daytime light exposure. In contrast, the advanced acrophase for the DST rhythm, the earlier bedtimes, and the shorter time interval (phase angle) between the DST rhythm and sleep–wake rhythm were all not associated with the overall daytime light exposure and physical activity in the older adults. Taken together, these results suggest that not environmental factors, but rather endogenous age-related changes of the circadian timing system might be primarily responsible for the phase advance of DST rhythm as well as for the altered phase relationship between the DST rhythm and sleep–wake rhythm in older adults. A possible explanation could be a rearrangement of internal phase angles among the circadian rhythms and/or a shortening of the endogenous period of the DST rhythm with aging that deserves further investigation. These results may provide a base for future studies investigating mechanisms responsible for age-related alterations in phase relationships between different circadian rhythms.

## 6. Limitations

This study has some limitations. First, the study examined only female participants because it was not possible to recruit a sufficient number of older men who were healthy, aged 60 years and over, and who agreed to participate in the study. This may limit the generalizability of the present findings, although a previous study showed that differences between sexes in DST were lost with aging [19]. A second limitation refers to the light meter integrated in the actigraph to measure ambient light. A recent study showed that the light meter has a good performance in monitoring the temporal pattern of light but may underestimate the absolute illuminance [71]. However, this would have affected both age groups equally in the present study and may therefore have no impact on group comparisons between older and young adults.

## Figures and Tables

**Table 1:** General characteristics of study participants.

Character	Older Women (*n* = 35)		Young Women (*n* = 30)		Group Comparison	
	Mean	SD	Mean	SD	t ^a^	*p*
Anthropometric characters						
Age (yrs)	68.94	4.94	25.63	3.64	39.64	<0.001
Weight (kg)	67.09	6.52	65.46	8.83	0.85	0.397
Height (m)	1.64	0.05	1.69	0.05	−4.50	<0.001
BMI (kg/m^2^)	24.99	2.69	22.76	2.64	3.36	0.001
Waist circumference (cm)	84.47	8.32	72.98	6.55	6.11	<0.001
Hip circumference (cm)	102.23	4.86	101.18	7.26	0.67	0.505
Waist–hip index	0.83	0.06	0.72	0.04	7.93	<0.001
Body composition ^b^						
Fat mass (kg)	23.40	6.37	20.96	6.37	1.53	0.132
Lean body mass (kg)	44.17	2.68	44.46	3.73	−0.36	0.718
Body cell mass (kg)	20.89	1.90	22.77	2.15	−3.71	<0.001
Basal metabolic rate (kcal/24 h)	1276	59.33	1335	66.94	−3.73	<0.001
Physical activity ^c,d^						
Total activity (counts), day	255,201	93,444	264,357	65,500	−0.45	0.654
Total activity (counts), night	21,470	53,269	14,003	5881	0.76	0.448
Light exposure ^c,d^						
Average light intensity (Lux), day	1019	1124	744	736	1.19	0.241
Average light intensity (Lux), night	15	70	12	42	0.19	0.851
Sleep–wake timing ^c,f^						
Awakening time (hh:mm) ^e^	06:35	45.09	07:35	37.28	−5.73	<0.001
Bedtime (hh:mm) ^e^	22:56	35.63	23:26	37.92	−3.34	0.001
Sleep-onset time (hh:mm) ^e^	23:22	43.11	23:43	38.57	−2.05	0.045
Sleep onset latency (min)	26.61	20.00	17.05	13.77	2.27	0.027
Total sleep time (h)	6.95	0.97	7.74	0.81	−3.49	0.001
WASO (min)	60.00	27.30	50.61	33.14	1.25	0.215
Sleep efficiency (%)	87.75	7.61	95.46	2.37	−4.81	<0.001

Abbreviations: BMI, body mass index; SD, standard deviation; WASO, waketime after sleep-onset; ^a^ *t*-test for independent groups; ^b^ N = 34 in older women; ^c^ averaged over seven days; ^d^ actigraphic data; ^e^ standard deviation is given in minutes; ^f^ data derived from actigraphy, corrected by data from sleep diary.

**Table 2:** Circadian characteristics of the rhythm of distal skin temperature in healthy older and young women, averaged over seven consecutive days.

Skin Temperature	Older Women (*n* = 35)		Young Women (*n* = 30)		ANOVA for Repeated Measures					
					Within Subject ^b^		Within Subject ^b^		Between Subjects ^c^	
					Older Women		Young Women			
	Mean	SEM	Mean	SEM	F	*p*	F	*p*	F	*p*
Hand (wrist)										
Mesor (°C)	33.11	0.11	33.21	0.10	2.26	0.073	0.52	0.730	0.04	0.844
Amplitude (°C)	1.16	0.10	1.44	0.09	0.68	0.664	0.64	0.701	4.17	0.046
Acrophase (hh:mm) ^a^	05:55	19.79	07:01	12.68	1.41	0.241	1.44	0.204	8.79	0.004
% Rhythm	68.84	1.57	67.42	1.97	-	-	-	-	-	-
Foot (heel)										
Mesor (°C)	31.87	0.10	32.12	0.15	0.65	0.630	0.64	0.652	0.21	0.151
Amplitude (°C)	1.80	0.09	2.20	0.15	0.39	0.845	2.02	0.066	0.54	0.023
Acrophase (hh:mm) ^a^	02:48	15.95	04:01	11.10	1.81	0.127	1.81	0.099	13.06	0.001
% Rhythm	84.56	1.17	82.78	1.18	-	-	-	-	-	-

SEM, standard error of the mean. ^a^ SEM is given in minutes; ^b^ comparison of seven days for reproducibility, separately for older and young women; ^c^ comparison of older and young women for age group differences.

**Table 3:** Relationship of circadian characteristics of distal skin temperature (amplitude and acrophase) and the phase angle with environmental light exposure, physical activity, and sleep–wake timing.

Character	Older Women (*n* = 35)						Young Women (*n* = 30)					
	Amplitude		Acrophase		Phase Angle ^a^		Amplitude		Acrophase		Phase Angle ^a^	
	r	*p*	r	*p*	r	*p*	r	*p*	r	*p*	r	*p*
Light exposure, Average light intensity (Lux) ^b,c^												
Hand, day	−0.42	0.023	0.30	0.106	0.33	0.072	−0.02	0.903	−0.10	0.608	−0.05	0.806
Foot, day	−0.45	0.007	0.00	0.999	0.07	0.678	0.03	0.867	0.04	0.850	0.12	0.523
Hand, night	−0.09	0.647	0.17	0.372	0.15	0.424	−0.04	0.838	−0.11	0.583	−0.20	0.299
Foot, night	−0.10	0.572	−0.06	0.753	−0.06	0.719	−0.01	0.964	−0.10	0.593	−0.23	0.217
Physical activity, Total activity (counts) ^b,c^												
Hand, day	−0.11	0.549	−0.33	0.080	−0.30	0.112	−0.21	0.276	−0.25	0.198	−0.37	0.053
Foot, day	−0.06	0.717	−0.29	0.091	−0.30	0.085	−0.24	0.204	−0.08	0.681	−0.24	0.207
Hand, night	−0.01	0.950	−0.15	0.428	−0.09	0.605	−0.12	0.558	−0.16	0.415	−0.10	0.606
Foot, night	−0.08	0.661	−0.01	0.954	0.03	0.878	−0.19	0.305	0.002	0.991	0.07	0.733
Awakening time (hh:mm) ^b,d^												
Hand	−0.05	0.802	−0.03	0.880	-	-	−0.27	0.181	0.42	0.031	-	-
Foot	0.06	0.728	0.31	0.074	-	-	0.24	0.213	0.56	0.002	-	-
Sleep-onset time (hh:mm) ^b,d^												
Hand	0.002	0.991	−0.11	0.548	-	-	−0.31	0.106	0.37	0.050	-	-
Foot	0.01	0.975	0.33	0.057	-	-	−0.03	0.888	0.59	0.001	-	-

^a^ Phase angle between the acrophase of distal skin temperature rhythm and the sleep-onset time of sleep–wake rhythm; ^b^ averaged over seven days; ^c^ actigraphic data; ^d^ data derived from actigraphy, corrected by data from sleep diary.

## Data Availability

The datasets presented in this article are not readily available because the participants of this study did not give written consent for their data to be shared publicly, due to the sensitive nature of the research.
